# T2T-YAO, T2T-SHUN, and more

**DOI:** 10.1016/j.gpb.2023.09.002

**Published:** 2023-09-22

**Authors:** Jingfa Xiao, Jun Yu

**Affiliations:** 1 National Genomics Data Center, Beijing Institute of Genomics, Chinese Academy of Sciences and China National Center for Bioinformation, Beijing 100101, China; 2 CAS Key Laboratory of Genome Sciences and Information, Beijing Institute of Genomics, Chinese Academy of Sciences and China National Center for Bioinformation, Beijing 100101, China; 3 University of Chinese Academy of Sciences, Beijing 100049, China

The first high-quality reference genome for Chinese populations has just been released [1], T2Y-YAO (尧yáo), and it starts a first-of-its-kind series for building virtual ancestor genomes (VAGs) for Chinese population-based studies and healthcare applications [2], [3]. The reasons are multifold. First, the Han people (汉人 hàn rén) are the world’s largest ethnicity, ∼ 1.5 billion in total, ∼ 19% of the global population, and 91% of the Chinese nationals. Second, the Chinese population history — regardless of whether the precise time period of each nation and State has or has not been determined — and complexity are being recorded faithfully in their genome sequences. Third, the Han ethnic groups are still co-habiting with other 55 minor ethnic peoples without geopolitical and geographical barriers.

In these obvious regards, we have yet to construct a reference genome-based network in which the edges and nodes are VAGs so connected based on between- and within-population sequence variations that are supported by largely within-population data (Figure 1). In other words, we need to choose historically representative geographical sites first and to assemble as many “nodes” — historically- or geographically-defined edge or reference genomes (HEGs) — as possible, and then to connect the neighboring HEGs to build a network. As the network and graphic elements grow in size and number, respectively, HEGs will be consolidated into VAGs. In the end, these VAGs will be tailored to the history of population migration and mixing, as well as their relevant timing, and most importantly, they can be used for effective genome-wide association study (GWAS) analyses for phenotype and disease/symptom mapping when the size of the inheritance-limited population reaches an expected significance. It is anticipated that thousands of HEGs and hundreds of VAGs are to be acquired for mapping useful variations that are disease- or health-relevant [4]. Therefore, we expect an effort to build such a network to be firmly supported within a few years [5], which serves as a database for population-centric studies as genome sequencing becomes affordable not only in rich counties but also in developing counties together with an awareness of the value of precision medicine to all human societies.

**Figure 1:**
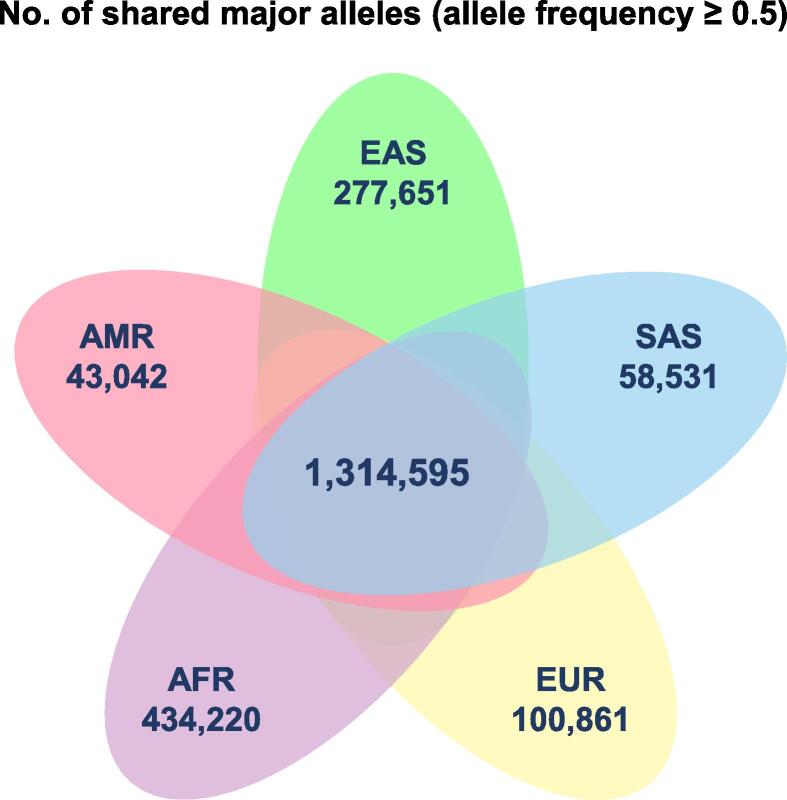
**Distribution of major alleles amongfivemajor ethnic groups** Data were extracted from the 1000 Genomes Project. The number indicates the count of major alleles (allele frequency ≥ 0.5) shared within each of the five major ethnic groups or among all of them. EAS, East Asian; AMR, admixed American; AFR, African; EUR, European; SAS, South Asian.

The purpose of expanding VAG-based network is to map genome variations effectively and precisely. One can imagine that an unknown genome sequence is better mapped to that of its close relatives rather than a distant one in which noises from between-population variations become substantially interruptive. Such a mapping process is often time-consuming, so it needs constant care for effectiveness and efficiency. Furthermore, within the next ten years or so, thousands and millions of genomes from most individuals will be acquired and the job of collecting, processing, and utilizing these data will definitely create enormous bottlenecks, let alone interpreting and explaining them to the doctors and the general public.

While we are welcoming the first HEG [we have yet to know if it will become one of the historically-defined ancestrial genomes (HAGs)], it is essential to point out the fact that not only T2T-SHUN (舜shùn) and T2T-YU (禹yǔ), where SHUN and YU are well-known Chinese ancestors different from YAO, are needed, but also several HEGs from each of the 55 Chinese ethnicities are to be expected!

## Competing interests

Both authors declare no competing interests.

## CRediT authorship contribution statement

**Jingfa Xiao:** Data curation, Writing – review & editing. **Jun Yu:** Conceptualization, Data curation, Writing – original draft, Writing – review & editing, Supervision. Both authors have read and approved the final manuscript.
